# Supplementary material for: Psychosocial Outcomes of Individuals Attending a Suicide Bereavement Peer Support Group: A Follow-Up Study
Source: Int J Environ Res Public Health. 2022 Mar 29;19(7):4076. doi: 10.3390/ijerph19074076 (PMC8998629; doi:10.3390/ijerph19074076)
Supplement: Supplementary file 1 [file ijerph-19-04076-s001.zip › ijerph-1644662-supplementary.pdf]

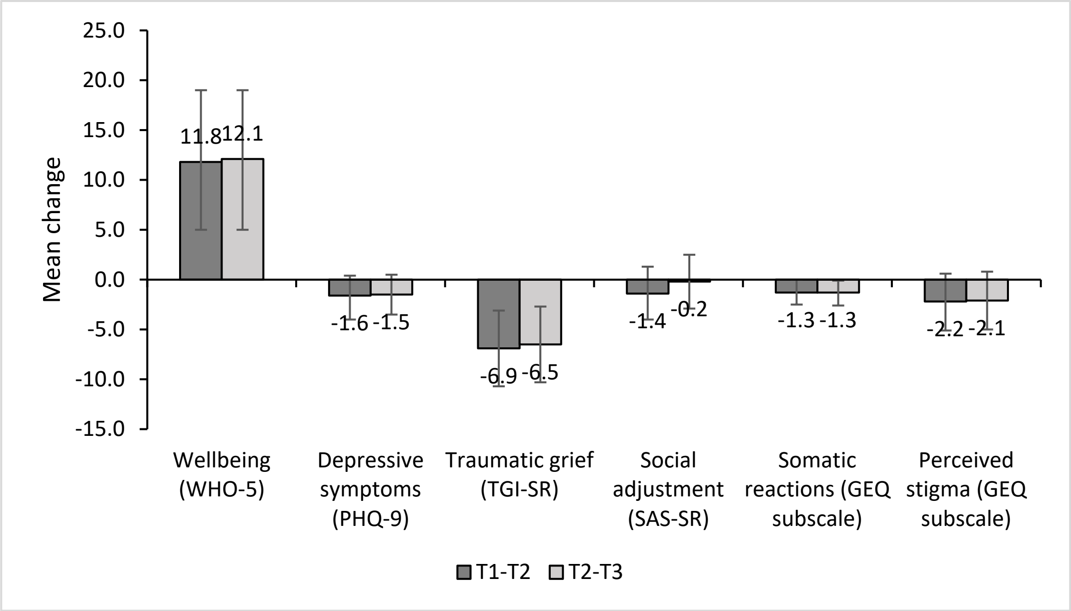

Figure S1. Changes in outcome measures at three-month (T2) and six-month (T3) follow-up.

Table S1. Change in suicidal ideation at three-month (T2) and six-month (T3) follow-up.

		**T2**		**T3**	
		**Reported Suicidal Ideation**	**No Suicidal Ideation Reported**	**Reported Suicidal Ideation**	**No Suicidal Ideation Reported**
**T1**	**Reported Suicidal Ideation**	5 (23.8%)	0 (0.0%)	5 (23.8%)	1 (4.8%)
	**No Suicidal Ideation Reported**	2 (9.5%)	14 (66.7%)	2 (9.5%)	13 61.9%)

T1-T2: p=0.500; T1-T3: p=1.000
